# Latent Tuberculosis Infection Is Associated with an Enrichment of Short-Chain Fatty Acid-Producing Bacteria in the Stool of Women Living with HIV

**DOI:** 10.3390/microorganisms12061048

**Published:** 2024-05-22

**Authors:** Suventha Moodley, Elouise Kroon, Charissa C. Naidoo, Georgina R. Nyawo, Benjamin G. Wu, Selisha Naidoo, Tinaye L. Chiyaka, Happy Tshivhula, Shivani Singh, Yonghua Li, Robin M. Warren, Eileen G. Hoal, Erwin Schurr, Jose C. Clemente, Leopoldo N. Segal, Marlo Möller, Grant Theron

**Affiliations:** 1 DSI-NRF Centre of Excellence for Biomedical Tuberculosis Research, SAMRC Centre for Tuberculosis Research, Division of Molecular Biology and Human Genetics, Faculty of Medicine and Health Sciences, Stellenbosch University, Tygerberg, Cape Town 7505, South Africa; suventha@sun.ac.za (S.M.); elouise_k@sun.ac.za (E.K.); ccnaidoo@sun.ac.za (C.C.N.); georginan@sun.ac.za (G.R.N.); selisha.naidoo@uct.ac.za (S.N.); tinayechiyaka@sun.ac.za (T.L.C.); tshivhula@sun.ac.za (H.T.); rw1@sun.ac.za (R.M.W.); egvh@sun.ac.za (E.G.H.); marlom@sun.ac.za (M.M.); 2 African Microbiome Institute, Division of Molecular Biology and Human Genetics, Department of Biomedical Sciences, Faculty of Medicine and Health Sciences, Stellenbosch University, Cape Town 7505, South Africa; 3 Division of Pulmonary and Critical Care Medicine, New York University Grossman School of Medicine, NYU Langone Health, New York, NY 10016, USA; benjamin.wu@nyulangone.org (B.G.W.); shivani.singh@nyulangone.org (S.S.); yonghua.li@nyulangone.org (Y.L.); leopoldo.segal@nyulangone.org (L.N.S.); 4 Department of Biochemistry, McGill University, Montreal, QC H3A 1Y6, Canada; erwin.schurr@mcgill.ca; 5 Program in Infectious Diseases and Immunity in Global Health, The Research Institute of the McGill University Health Centre, 1001 Boul Décarie, Site Glen Block E, Room EM3.3210, Montréal, QC H4A 3J1, Canada; 6 McGill International TB Centre, McGill University, Montréal, QC H3A3J1, Canada; 7 Departments of Medicine and Human Genetics, McGill University, Montréal, QC H3A0C7, Canada; 8 Department of Genetics and Genomic Sciences, Icahn School of Medicine at Mount Sinai, New York, NY 10029, USA; jose.clemente@mssm.edu

**Keywords:** microbiota, latent tuberculosis, HIV, short-chain fatty acids

## Abstract

Latent tuberculosis infection (LTBI) is common in people living with HIV (PLHIV) in high-TB-burden settings. Active TB is associated with specific stool taxa; however, little is known about the stool microbiota and LTBI in PLHIV. We characterised the stool microbiota of PLHIV with [interferon-*γ* release assay (IGRA)- and tuberculin skin test (TST)-positive] or without (IGRA- and TST-negative) LTBI (*n* = 25 per group). The 16S rRNA DNA sequences were analysed using QIIME2, Dirichlet-Multinomial Mixtures, DESeq2, and PICRUSt2. No α- or β-diversity differences occurred by LTBI status; however, LTBI-positive people were *Faecalibacterium*-, *Blautia*-, *Gemmiger*-, and *Bacteroides*-enriched and *Moryella*-, *Atopobium*-, *Corynebacterium*-, and *Streptococcus*-depleted. Inferred metagenome data showed that LTBI-negative-enriched pathways included several metabolite degradation pathways. Stool from LTBI-positive people demonstrated differential taxa abundance based on a quantitative response to antigen stimulation. In LTBI-positive people, older people had different β-diversities than younger people, whereas in LTBI-negative people, no differences occurred across age groups. Amongst female PLHIV, those with LTBI were, vs. those without LTBI, *Faecalibacterium*-, *Blautia*-, *Gemmiger*-, and *Bacteriodes*-enriched, which are producers of short-chain fatty acids. Taxonomic differences amongst people with LTBI occurred according to quantitative response to antigen stimulation and age. These data enhance our understanding of the microbiome’s potential role in LTBI.

## 1. Introduction

Tuberculosis (TB) is a major cause of death, with 167,000 deaths among persons living with HIV (PLHIV) in 2022 [1]. One strategy to prevent active TB is to control latent tuberculosis infection (LTBI). LTBI is inferred from a positive tuberculin skin test (TST) or interferon-gamma release assay (IGRA). TB preventive treatment (TPT) strategies play a key role in TB prevention especially in vulnerable populations such as PLHIV. PLHIV are at greater risk for *Mycobacterium tuberculosis* (MTB) infection and progression to active TB [2]. However, it is poorly understood why some individuals in TB-endemic countries are never infected despite high exposure and why a large proportion of infected individuals never progress [3,4]. We need more information on the correlates of infection and progression, which may have prognostic value.

The microbiome has important immunomodulating effects, and the microbiome’s role, including in people with LTBI, is an emerging area of interest. For example, in the lung, *Lactobacillus* is enriched in people with LTBI compared to the active pulmonary TB group and LTBI-negative people [5]. In the nasopharynx of LTBI-positive people, *Staphylococcus* and *Corynebacterium* dominate the microbiome compared to healthy control and active TB cases [6], and the nasopharyngeal microbiota of LTBI-positive people has lower alpha-diversity than that of LTBI-negative people [7]. The stool microbiota has potentially an important immunomodulatory role in respiratory disease, including active TB [8]. However, it is comparatively understudied in latent TB. One study among individuals with poorly controlled diabetes showed LTBI-positive people to be *Bacteroides*-, *Alistipes*-, and *Blautia*-enriched compared to LTBI-negative people [9]. During LTBI infection, comparisons of TB cases, HIV-negative LTBI-positive individuals, and LTBI-negative and active TB gut microbiomes showed trends of changes in *Bacteroides* and Firmicutes; however, no significant difference was observed in the composition of the stool microbiota [10]. HIV-negative LTBI-positive individuals showed a positive correlation between relative abundances of *Coriobacteriaceae* and IFN-gamma against MTB antigens more likely associated with CD4+ T cells [11]. A key knowledge gap still exists because those studies did not include PLHIV. Not only is HIV associated with reductions in the diversity in the stool microbiota [12], but PLHIV also have higher rates of TB infection and progression. If microbial dysbiosis is detected early after exposure, it may be indicative of early microbial and immune dysregulation associated with incident TB. Biomarkers of progression to active TB are a major public health priority, as is understanding the biological drivers of LTBI and progression. Therefore, to address these knowledge gaps, we evaluated the stool microbiota of PLHIV with and without LTBI. We hypothesised that taxonomic differences will be seen between PLHIV with and without LTBI.

## 2. Methods

### 2.1. Recruitment

Participants (18–60 years) were recruited from community health care clinics in Cape Town, South Africa, as part of a published parent study (ResisTB) [13]. This cohort was predominantly female, and age was a surrogate for TB exposure, resulting in two groups of 18–25 years and 35–60 years. The participants were recruited in the Western Cape, a high-TB-incidence area where 80–90% of the population between 31 and 35 years old display TST reactions >10 mm [14,15]. The parent study used age as a surrogate for cumulative exposure, recruiting adults with extreme ages into two groups (18–25 or 35–60 years old). All people had to be TB-symptom-screen-negative, HIV-positive, and stable on ART for ≥1 year. Study procedures were approved by the Stellenbosch University Human Research Ethics Committee (N16/03/033A), and each participant provided written informed consent.

### 2.2. Definitions

IGRA- and TST-positive (LTBI-positive) people were defined by two positive QuantiFERON-TB Gold Plus tests and a positive TST (>0 mm). IGRA- and TST-negative (LTBI-negative) people were defined by two negative QuantiFERON-TB Gold Plus tests and a TST (0 mm).

### 2.3. Microbiota Specimen Collection and Processing

At TST administration, participants were provided with a home stool sampling kit containing an EasySampler (ALPCO, Salem, NH, USA) and a receptacle containing DNA stabilisation buffer (Stratec Biomedical, Birkenfeld, Germany). Generally, buffered stools were collected the night before TST reading and returned at TST reading. Upon receipt at the laboratory, buffered stool was frozen at −20 °C until batched DNA extraction was carried out using the PSP Spin Stool DNA Plus Kit (Stratec Biomedical, Birkenfeld, Germany).

### 2.4. 16S rRNA Gene Sequencing and Microbiota Analysis

V4 region sequencing of the bacterial 16S rRNA gene (150 bp read length, paired-end) was conducted using Illumina Miseq (Illumina, San Diego, CA, USA), as described in [8]. Sequences was analysed with Quantitative Insights into Microbial Ecology (QIIME2, version 2.0.8). Cluster analysis was carried out using Dirichlet-Multinomial Mixtures (DMM) [16]. Alpha-diversity was calculated by Shannon’s diversity with Mann–Whitney testing using GraphPad Prism (v8 GraphPad Software, Boston, MA, USA). Beta-diversity was calculated using Bray–Curtis with permutational multivariate ANOVA (PERMANOVA) using R (v4.2.2). Functional metagenome was inferred from sequencing data using Phylogenetic Investigation of Communities by Reconstruction of Unobserved States (PICRUSt2; V.2.1.3-b) [17]. Differentially abundant taxa and metabolic pathways were identified using *DESeq2* (v1.22.2) [18] and Benjamini–Hochberg correction adjustment for multiple comparisons (significance level 0.20) [8,19]. For comparison groups, taxa at higher relative abundance in one group were described as enriched (those at lower relative abundance were described as depleted). For the whole cohort, age and field site correction was applied for *DESeq2* analyses. Linear discriminant analysis (LDA) effect size (LEfSe) [20] was used to compare the clusters to each other. The proportions test was performed using STATA (v18; StataCorp, College Station, TX, USA) to determine whether a specific variable was more frequent in different groups.

## 3. Results

### 3.1. Population

**Figure 1:**
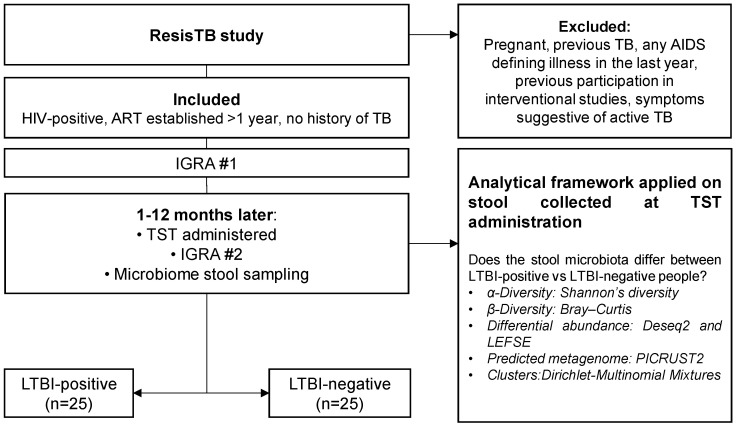
Study flow diagram. We collected and analysed stool from 25 LTBI-positive and 25 LTBI-negative people enrolled in a parent study (ResisTB). Abbreviations: ART: antiretroviral therapy; HIV: human immunodeficiency virus; IGRA: interferon-gamma release assay; TST: tuberculin skin test; LTBI: latent TB infection; TB: tuberculosis.

We collected stool from female PLHIV stable on ART who did not have previous TB, with or without LTBI (*n* = 25 per group; Figure 1). Demographic data are shown in Table 1. People with LTBI were younger and more likely to be from Khayelitsha (Site B) Community Health Clinic (Table 1).

### 3.2. Stool from People with LTBI Is Moryella-, Atopobium-, Corynebacterium-, and Streptococcus-Depleted and Faecalibacterium-, Blautia-, Gemmiger-, and Bacteroides-Enriched

**Figure 2:**
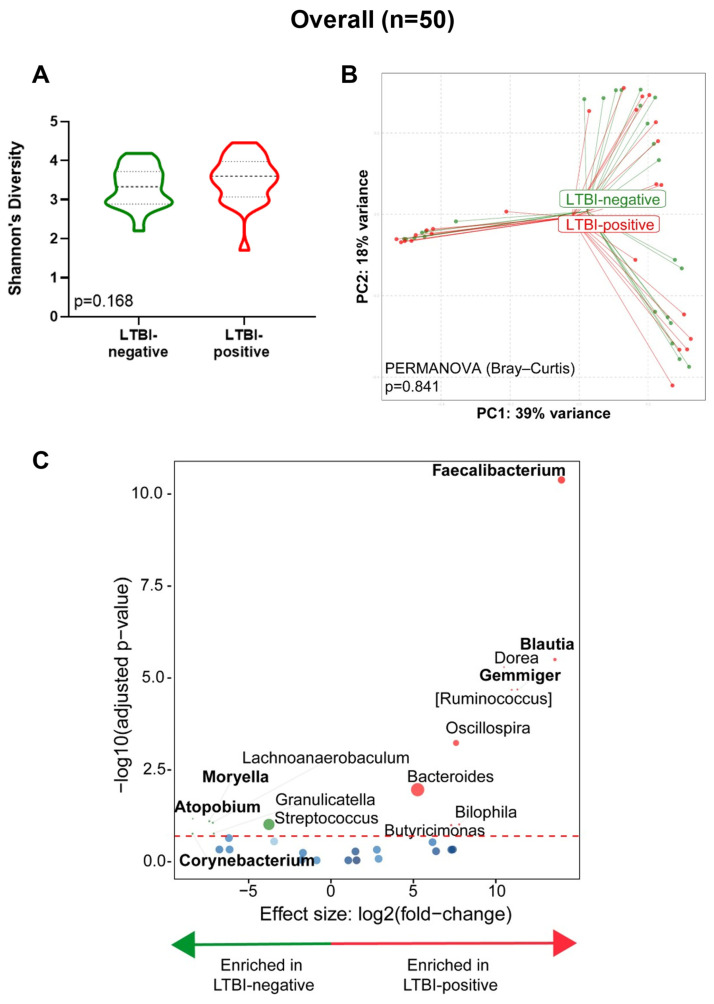
Stool from LTBI-positive people is *Moryella*-, *Atopobium*-, *Corynebacterium*-, and *Streptococcus*-depleted and *Faecalibacterium*-, *Blautia*-, *Gemmiger*-, and *Bacteroides*-enriched. (**A**) Comparison of Shannon’s diversity index of LTBI-positive and LTBI-negative groups. (**B**) Principal coordinate analysis of Bray–Curtis distances between groups. (**C**) Volcano plot depicting differentially abundant taxa. More discriminatory taxa (bolded) appear closer to the left or right and higher above the threshold (red dotted line, FDR = 0.20). Relative abundance of taxa is indicated by circle size. The blue circles are taxa below the selected threshold. Abbreviation: LTBI: latent TB infection.

Overall, no differences were seen by LTBI status for α- (*p* = 0.168, Figure 2A) and β-diversity (PERMANOVA, *p* = 0.841, Figure 2B). However, *Moryella*, *Atopobium*, *Corynebacterium*, and *Streptococcus* were depleted and *Faecalibacterium*, *Blautia*, *Gemmiger*, *and Bacteroides* were enriched in LTBI-positive people compared to LTBI-negative people (Figure 2C). People on TB preventative treatment (INH prophylaxis) were, compared to those not on treatment, *Blautia*-enriched and *Moraxella*-, *Megamonas*-, and *Actinobacillus*-depleted (Supplementary Figure S1).

### 3.3. Comparisons According to Age Groups (18–25 vs. 35–60 Years)

**Figure 3:**
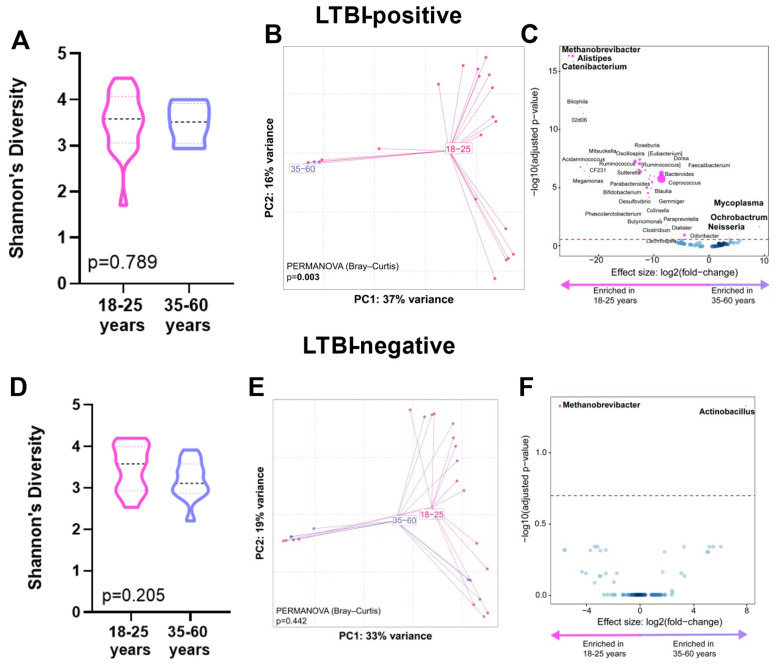
Distinct stool microbiotas by age in LTBI-positive people. Shannon’s diversity index, principal coordinate analysis of Bray–Curtis distances, and volcano plot depicting differentially abundant taxa enriched in 35–60 and 18–25 age groups are for LTBI-positive people only (**A**–**C**) and for LTBI-negative people only (**D**–**F**). More discriminatory taxa (bolded) appear closer to the left or right and higher above the threshold (red dotted line, FDR = 0.20). Relative abundance of taxa is indicated by circle size. The blue circles are taxa below the selected threshold. Abbreviation: LTBI: latent TB infection.

When we compared age groups within people of the same LTBI status, within LTBI-positive individuals, no α-diversity differences occurred (*p* = 0.789, Figure 3A); however, the β-diversity differed (*p* = 0.003, Figure 3B). Older LTBI-positive people were *Ochorobactrum*-, *Neisseria*-, *and Mycoplasma*-enriched and *Catenibacterium*-, *Alistipes*-, and *Methanobrevibacter*-depleted (Figure 3C). LTBI-negative individuals did not differ in α-diversity (*p* = 0.205, Figure 3D) or β-diversity (*p* = 0.442, Figure 3E), and older LTBI-negative people were also *Methanobrevibacter*-depleted and *Actinobacillus*-enriched. (Figure 3F). We calculated the Bray–Curtis distances for older vs. younger LTBI-positive and LTBI-negative people. Both age groups demonstrated similar distances (*p* = 0.952) across LTBI statuses (Supplementary Figure S2).

### 3.4. Distinct Metabolic Pathway Associations with LTBI-Positive and -Negative People

**Figure 4:**
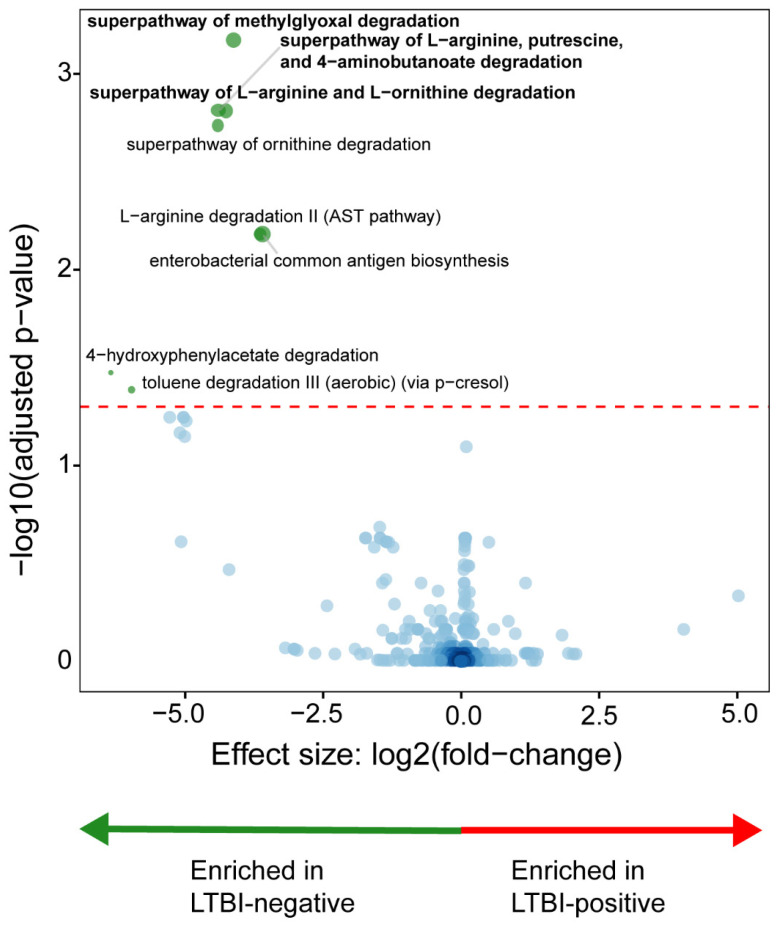
Distinct microbial metabolic pathways are associated with LTBI. LTBI-positive people had a depletion of degradation-associated pathways. More discriminatory pathways (bolded) appear closer to the left or right and higher above the threshold (red dotted line, FDR = 0.20). The blue circles are pathways below the selected threshold. Abbreviation: LTBI: latent TB infection.

LTBI-negative people were enriched in the methylglyoxal, L-arginine, putrescine, 4-aminobutanoate, and L-ornithine degradation pathways (Figure 4). LTBI-positive people had no differential enrichment.

### 3.5. Microbial Cluster Identification and Their Characteristics

**Figure 5:**
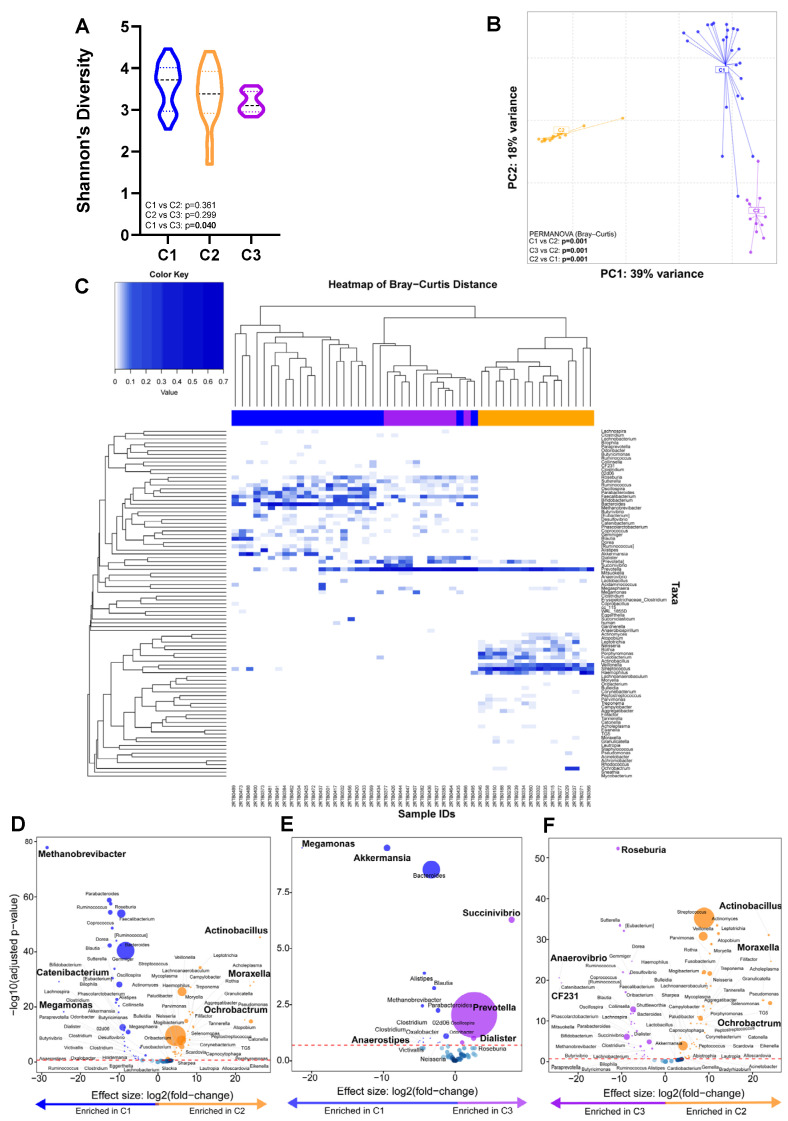
Three distinct microbial clusters with a high abundance of *Bacteroides*, *Streptococcus*, and *Prevotella* were identified. Comparison of (**A**) alpha- and (**B**) beta-diversity by cluster. (**C**) Heatmap shows the composition of each cluster. (**D**–**F**) Volcano plots depicting differentially abundant taxa compared across cluster pairs. More discriminatory taxa (bolded) appear closer to the left or right and higher above the threshold (red dotted line, FDR = 0.20). The blue circles are pathways below the selected threshold. Abbreviation: LTBI: latent TB infection.

*Taxonomic analyses:* Three clusters (C1, C2, and C3) were identified (Supplementary Figure S3) with C1 vs. C2 (*p* = 0.361) and C2 vs. C3 (*p* = 0.299) having similar α-diversity whilst C1 vs. C3 differed (*p* = 0.040) (Figure 5A). The β-diversity differed between clusters (PERMANOVA *p* = 0.001) (Figure 5B). C1, C2, and C3 were characterised by high *Bacteriodes*, *Streptococcus*, and *Prevotella* abundances, respectively (Figure 5C). When cluster pairs were compared (Figure 5D–F), C1 was, compared to C2, *Methanobrevibacter*-, *Catenibacterium*-, and *Megamonas*-enriched and *Actinobacillus*-, *Moraxella*-, and *Ochrobactrum*-depleted and, compared to C3, *Megamonas*-, *Akkermansia*-, and *Anaerostipes*-enriched and *Succinivibrio*-, *Prevotella*-, and *Dialister*-depleted. C3 was, compared to C2, *Roseburia*-, *Anaerovibrio*-, and *CF231*-enriched and *Actinobacillus*-, *Moraxella-*, and *Ochrobactrum*-depleted. When all three clusters were compared together (Supplementary Figure S4), C1 was, relative to the others, the most enriched in *Bacteroides*, *Oscillospirai*, and *Parabacteroides.* C2 was the most enriched in *Streptococcus*, *Veillonella*, and *Actinomyces*, and C3 was the most enriched in *Prevotella* and *Catenibacterium*.

*Clinical and demographic characteristics across clusters:* β-diversity differed by field site (Supplementary Table S1). There were no differences in the proportion of LTBI-positive people per cluster (Table 2). People in C1 and C3 were more likely to be on INH prophylaxis than C2 and more likely to be from sites other than Khayelitsha (Site B) Youth. People in C2 were, compared to those in C3, more likely to be from sites other than Khayelitsha (Site B) CHC Youth and Du Noon CDC.

### 3.6. Within LTBI-Positive People, Taxa Are Differentially Enriched Based on the Magnitude of the Response to Antigen Stimulation

**Figure 6:**
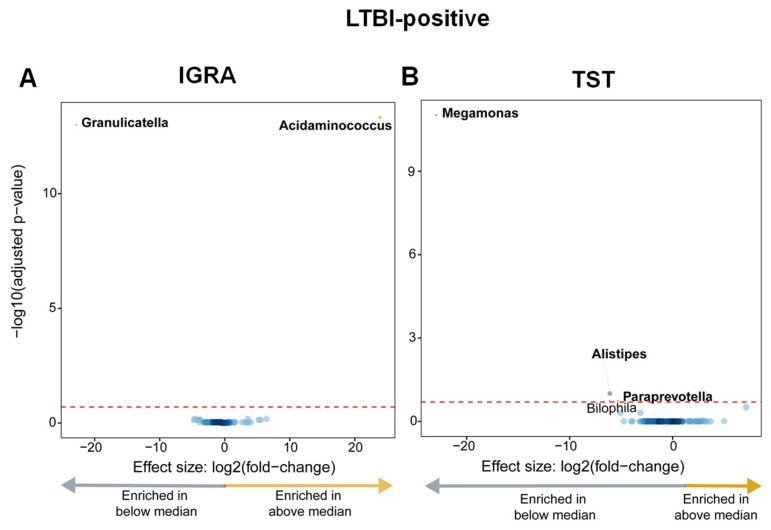
LTBI-positive people had, based on the magnitude of the quantitative responses, differentially enriched taxa (those with IGRA responses above the median were *Acidaminococcus*-enriched and *Granulicatella*-depleted; those with TST responses below the median were *Megamonas*-, *Alistipes*-, and *Paraprevotella*-enriched). (**A**) Volcano plot depicting differentially abundant taxa based on the median IGRA quantitative response to antigen (median value 5.56). More discriminatory taxa appear closer to the left or right and higher above the threshold (red dotted line, FDR = 0.20). The blue circles are pathways below the selected threshold. (**B**) Volcano plot depicting differentially abundant taxa based on the median TST response (median value: 18). Abbreviation: LTBI: latent TB infection.

Taxonomic abundances between quantitative responses to antigen stimulation were compared overall and in LTBI-positive people. No differential abundances were identified between the overall cohort above or vs. the median average IGRA or TST quantitative response (Supplementary Figure S5A,B). LTBI-positive people with an IGRA response above the median were *Acidaminococcus*-enriched and *Granulicatella*-depleted (Figure 6A), and LTBI-positive people below the median TST response were *Megamonas*-, *Alistipes*-, and *Paraprevotella*-enriched (Figure 6B).

## 4. Discussion

We compared, in PLHIV, the stool microbiota of LTBI-positive vs. LTBI-negative women. Our key findings are as follows: (1) the stool from LTBI-positive people differed from that of LTBI-negative people in terms of known short-chain fatty acid (SCFA)-producing taxa, and these were associated with a depletion of metabolite degradation pathways; (2) three taxonomic clusters occurred, characterised by high abundances of *Bacteroides*, *Streptococcus*, and *Prevotella* and clustering associated with INH prophylaxis and health facility location; (3) LTBI-positive people with a greater quantitative response to mycobacterial antigen stimulation were, vs. LTBI-positive people with lesser responses, *Acidaminococcus*-enriched (IGRA readouts) and *Megamonas*-, *Alistipes*-, and *Paraprevotella*-depleted (TST); and (4) the β-diversity differed by age group only in LTBI-positive people. Our findings help lay a foundation for understanding the microbiome’s role in LTBI. Stool from people with LTBI was *Moryella*-, *Atopobium*-, *Corynebacterium*-, and *Streptococcus*-depleted and *Faecalibacterium*-, *Blautia*-, *Gemmiger*-, and *Bacteroides*-enriched. *Faecalibacterium* and *Gemmiger* are known producers of butyrate [21], which is an SCFA that increases incident TB risk [22]. *Bacteroides* produces SCFAs like acetate and propionate [23]. *Blautia* is enriched in people with active TB and independently predicts the upregulation of proinflammatory pathways [8]. Although the role of *Atopobium* is unclear, *Streptococcus*, which we found to be depleted in LTBI-positive people, produces acetate, which mitigates host inflammation [24].

Three taxonomic clusters occurred [*Bacteroides* (C1), *Streptococcus* (C2), *Prevotella*-enriched (C3)]; however, these were not associated with LTBI status (other studies have documented specific clusters associated with active TB [19]). People within each cluster were more likely to be recruited from different facilities, suggesting potential geographic associations to be considered in future studies. Additionally, C1 was more likely than C2 to be receiving INH prophylaxis. INH prophylaxis itself was associated with *Blautia* enrichment and *Moraxella*, *Megamonas*, and *Actinobacillus* depletion. Other studies have shown *Clostridiales*-, *Coprococcus*-, *Lachnospiraceae*-, and *Ruminococcaceae*-enriched and *Clostridium*_XIVa-, *Romboutsia*-, and *Roseburia*-depleted stool to occur during rifamycin-based tuberculosis preventive therapy [25]. To our knowledge, our study is the first to show *Blautia*-enriched and *Moraxella*-, *Megamonas*-, and *Actinobacillus*-depleted stool in humans on isoniazid TB preventive therapy. This association is interesting as isoniazid itself is a drug thought to have an extremely narrow antimicrobial spectrum (*Mycobacteria* only) [26].

Additionally, LTBI-positive people who had a larger quantitative response to antigen stimulation were, when IGRA readouts were used, *Acidaminococcus*-enriched and *Granulicatella*-depleted and, when TST readouts were used, *Megamonas*-, *Alistipes*-, and *Paraprevotella*-depleted. *Acidaminococcus* produces acetate and butyrate [27] and is primarily influenced by diet [28]; its enrichment likely reflects lifestyle differences within LTBI-positive people. *Paraprevotella* (like *Alistipes*) is an SCFA producer and is generally considered beneficial [29]. This finding is notable because higher quantitative responses are associated with a greater risk of incident TB [30], suggesting such taxa may contribute to this risk; however, this requires prospective confirmation in controlled studies. Younger people were more likely to be LTBI-positive than older people, so, to adjust for age as a potential confounder, we dichotomised people (18–25 and 35–60 years). Within LTBI-positive people, older vs. younger individuals were enriched in *Ochrobactrum* (an opportunistic pathogen [31]), *Neisseria* (role unclear), and *Mycoplasma* (induces a proinflammatory cytokines [32]) and depleted in *Catenibacterium* (enriched in PLHIV [33] and active TB [34]), *Alistipes* (SCFA producer with potentially anti-inflammatory effects [35]), and *Methanobrevibacter* (a methane producer [36]). Older LTBI-negative people were also *Methanobrevibacter*-depleted and enriched in *Actinobacillus* (inversely associated with amino acid production [37]) but did not show β-diversity differences. This could suggest that LTBI results in greater age-related microbiome differences but requires further investigation.

Our study has strengths and limitations. This is a cross-sectional study that, to enhance feasibility, leveraged (but was constrained to) the parent ResisTB study. We only evaluated women with HIV, and other populations may result in different findings; however, PLHIV do have an elevated risk of incident TB. People aged 26–34 were not recruited by the parent study and were hence unavailable to us. This may affect our findings. Although people were measured once, our study generates useful data to inform hypothesis-driven interventions to potentially modulate the microbiome. In conclusion, amongst women living with HIV, those with LTBI were, vs. those without LTBI, primarily differentially abundant in SCFA-producing anaerobic bacteria. Taxonomic differences also occurred amongst people with LTBI by age group, suggesting that age-related microbiome perturbations are more pronounced in LTBI-positive people. Whether the taxonomic associations with TB infection identified here influence or play a casual role in TB progression requires future research. Longitudinal studies are needed to further delineate the microbiome’s role in LTBI, which this work helps provide a justification for.

## Figures and Tables

**Table 1:** Demographic and clinical characteristics. People with LTBI were younger and more likely to from Khayelitsha (Site B) CHC. Abbreviations: LTBI: latent TB infection; BMI: body mass index; INH: isoniazid; CHC: Community Health Centre; CDC: Community Day Centre. Data are median (IQR) or *n* (%).

Characteristic *	Overall (*n* = 50)	LTBI-Positive (*n* = 25)	LTBI-Negative (*n* = 25)	*p*-Value
Age, years	24 (23–39)	24 (22–25)	38 (24–41)	**0.005**
18–25	33/50 (66)	21/25 (84)	12/25 (48)	
35–60	17/50 (34)	4/25 (16)	13/25 (52)	**0.007**
CD4 (cells/mm^3^)	468 (368–683)	471 (346–675)	434 (375–705)	0.881
BMI (kg/m)	29 (25–33)	30 (28–34)	26 (24–32)	0.137
Current Tobacco smoker	1/49 (2)	0/24 (0)	1/25 (4)	0.312
Alcohol	31/50 (62)	17/25 (68)	14/25 (56)	0.382
INH prophylaxis	12/50 (24)	6/25 (24)	6/25 (24)	>0.999
Co-trimoxazole prophylaxis	7/50 (14)	2/25 (8)	5/25 (20)	0.221
Field site				
Khayelitsha (Site B) CHC	10/50 (20)	8/25 (32)	2/25 (8)	**0.034**
Khayelitsha (Site B) Youth	7/50 (14)	4/25 (16)	3/25 (12)	0.684
Kraaifontein CHC	11/50 (22)	1/25 (4)	10/25 (40)	**0.002**
Site C Youth	19/50 (38)	12/25 (48)	7/25 (28)	0.145
Du Noon CDC	3/50 (12)	0/25 (0)	3/25 (12)	0.074

Bolded *p* values are significant at *p* < 0.05. * Missing data: current Tobacco smoker (*n* = 1).

**Table 2:** Demographic and clinical characteristics of the three clusters found in cohort. C1 was more likely than C2 to be on current INH prophylaxis and more likely to be recruited from Khayelitsha (Site B) CHC, Kraaifontein CHC, Site C Youth, or Du Noon CDC. C2 was more likely than C3 to be recruited from Khayelitsha (Site B) CHC, Kraaifontein CHC, or Site C Youth. Abbreviations: LTBI: latent TB infection; BMI: body mass index; INH: isoniazid; CHC: Community Health Centre; CDC: Community Day Centre. Data are median (IQR) or *n* (%).

Characteristics *	C1 (*n* = 23)	C2 (*n* = 16)	C3 (*n* = 11)	*p*-Value C1 vs. C2	*p*-Value C2 vs. C3	*p*-Value C1 vs. C3
Age, years	24 (22–25)	37 (24–40)	25 (24–33)	0.114	0.723	0.146
LTBI-positive	11/23 (48)	9/16 (56)	5/11 (46)	0.601	0.581	0.900
CD4 (cells/mm^3^)	575 (375–700)	395 (372–516)	487 (317–642)	0.242	0.914	0.445
BMI (kg/m)	29 (25–32)	30 (28–35)	27 (23–34)	0.248	0.277	0.612
Current Tobacco smoker	0/23 (0)	0/15 (0)	1/11 (9)	-	0.234	0.142
Alcohol	15/23 (65)	9/16 (56)	7/11 (64)	0.574	0.704	0.927
INH prophylaxis	8/23 (35)	1/16 (6)	3/11 (27)	**0.037**	0.130	0.662
Co-trimoxazole prophylaxis	3/23 (13)	4/16 (25)	0/11 (0)	0.337	0.072	0.211
Field site						
Khayelitsha (Site B) CHC	0/23 (0)	10/16 (63)	0/11 (0)	**<0.001**	**<0.001**	-
Khayelitsha (Site B) Youth	1/23 (4)	3/16 (19)	3/11 (27)	0.1440	0.597	0.051
Kraaifontein CHC	7/23 (30)	0/16 (0)	4/11 (36)	**0.014**	**0.009**	0.726
Site C Youth	15/23 (65)	0/16 (0)	4/11 (36)	**<0.001**	**0.009**	0.114
Du Noon CDC	0/23 (0)	3/16 (19)	0/11 (0)	**0.030**	0.127	-

Bolded items indicate that *p* values are significant at *p* < 0.05. * Missing data: current Tobacco smoker (*n* = 1).

## Data Availability

Sequences are available in the Sequence Read Archive (PRJNA1113869). Data and R scripts used for analyses are publicly available upon publication. The original contributions presented in the study are included in the article/Supplementary Material, further inquiries can be directed to the corresponding author.

## Supplementary Materials

The following supporting information can be downloaded at: https://www.mdpi.com/article/10.3390/microorganisms12061048/s1, Figure S1: People who received INH prophylaxis were *Blautia*-enriched and *Moraxella*-, *Megamonas*- and *Actinobacillus*-depleted. Volcano plot depicting differentially abundant taxa based on INH prophylaxis. More discriminatory taxa appear closer to the left or right, and higher above the threshold (red dotted line, FDR = 0.20). Figure S2. Bray–Curtis distances between 35–60 and 18–25 in LTBI-positives vs LTBI-negatives are similar. Bray–Curtis distances between 35-60 and 18–25 in LTBI-positives vs LTBI-negatives, where similar distances indicate less dissimilarity. LTBI: Latent TB infection. Figure S3: The DMM LaPlace approximation. Three clusters were identified as the best fit using Dirichlet-Multinomial Mixtures (DMM) in the stool samples collected in the overall cohort. Figure S4: Taxonomic differences between the three clusters found in the cohort. (A) Linear discriminant analysis (LDA) effect size (LEfSe) identified significant taxonomic differences in microbiome enrichment based on clusters. (B) Relative abundance of each genus. Figure S5: No difference between taxa associated with quantitative response to antigen stimulation. (A) Volcano plot depicting differentially abundant taxa based on the median IGRA quantitative response to antigen (median value 0.43). More discriminatory taxa appear closer to the left or right, and higher above the threshold (red dotted line, FDR = 0.20). (B) Volcano plot depicting differentially abundant taxa based on the median TST response (Median value 25). LTBI: Latent TB infection. Table S1: Alpha and Beta diversity compared by cohort characteristics. In addition to the age differences seen in Figure 2, β-diversity differences were seen between field sites.
