# Supplementary material for: Latent Tuberculosis Infection Is Associated with an Enrichment of Short-Chain Fatty Acid-Producing Bacteria in the Stool of Women Living with HIV
Source: Microorganisms. 2024 May 22;12(6):1048. doi: 10.3390/microorganisms12061048 (PMC11205370; doi:10.3390/microorganisms12061048)

Supplementary Material

Results 2

Supplementary Figure S1: People who received INH prophylaxis were *Blautia*-enriched and *Moraxella*-, *Megamonas*-, and *Actinobacillus*-depleted 3

Supplementary Figure S2. Bray–Curtis distances between 35-60 and 18-25 age groups in LTBI-positive vs. LTBI-negative people are similar 4

Supplementary Table S1: Alpha- and beta-diversity compared by cohort characteristics 5

Supplementary Figure S3: The DMM LaPlace approximation. 6

Supplementary Figure S4: Taxonomic differences between the three clusters found in the cohort. 7

Supplementary Figure S5: No difference between taxa associated with quantitative response to antigen stimulation 8

Results

Taxa associated with INH prophylaxis

The differential taxonomic abundance between the participants that received INH prophylaxis was tested using *DESeq2*. People who received INH prophylaxis were *Blautia*-enriched and *Moraxella*-, *Megamonas*-, and *Actinobacillus*-depleted (**Supplementary Figure S1**).

α- and β-diversities according to demographic and clinical characteristics

Bray–Curtis distances between 35-60 and 18-25 age groups in LTBI-positive vs. LTBI-negative people were compared (**Supplementary Figure S2**). Alpha-diversity was calculated by Shannon's diversity with Kruskal–Wallis testing in R (v4.2.2; R Core Team). Beta-diversity was calculated using Bray–Curtis with permutational multivariate ANOVA (PERMANOVA) in R (v4.2.2; R Core Team). In the overall cohort, β-diversity differences were seen between field sites overall, but no differences were seen in LTBI-positive and LTBI-negative people **(Supplementary Table S1)**.

The DMM LaPlace approximation

Three clusters were identified as the best fit using Dirichlet-Multinomial Mixtures (DMMs) in the stool samples collected in the overall cohort **(Supplementary Figure S2)**.

Linear discriminant analysis (LDA) effect size (LEfSe) comparing all three clusters

When all three clusters were compared together (**Supplementary Figure S4**), C1 was, relative to the others, the most enriched in *Bacteroides*, *Oscillospirai*, and *Parabacteroides*. C2 was the most enriched in *Streptococcus*, *Veillonella*, and *Actinomyces*, and C3 was the most enriched in *Prevotella* and *Catenibacterium*.

Taxa associated with quantitative response to antigen stimulation

The differential taxonomic abundance between quantitative responses to antigen stimulation was tested using *DESeq2*. There were no differences between taxa associated with quantitative response to antigen stimulation in the overall cohort **(Supplementary Figure S5).**

Supplementary Figure S1: People who received INH prophylaxis were *Blautia*-enriched and *Moraxella*-, *Megamonas*-, and *Actinobacillus*-depleted. Volcano plot depicting differentially abundant taxa based on INH prophylaxis. More discriminatory taxa appear closer to the left or right and higher above the threshold (red dotted line, FDR=0.20).

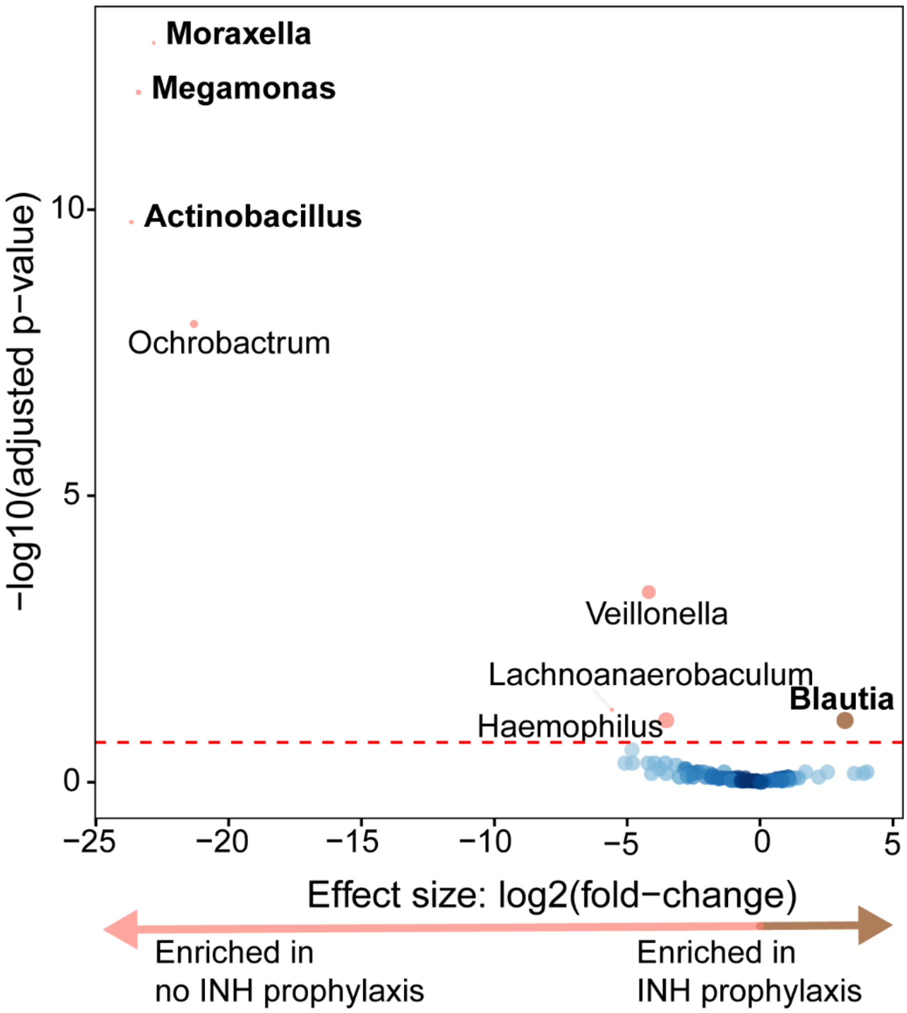

Supplementary Figure S2. Bray–Curtis distances between 35-60 and 18-25 age groups in LTBI-positive vs. LTBI-negative people are similar. Bray–Curtis distances between 35-60 and 18-25 age groups in LTBI-positive vs. LTBI-negative people, where similar distances indicate less dissimilarity. LTBI: latent TB infection.

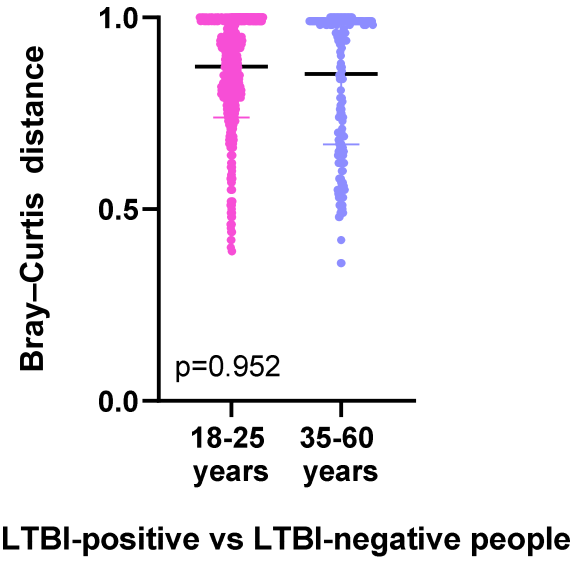

Supplementary Table S1: Alpha- and beta-diversity compared by cohort characteristics. In addition to the age differences seen in Figure 2, β-diversity differences were seen between field sites.

**Characteristic**	**Overall (n=50)**			**LTBI-positive (n=25)**			**LTBI-negative (n=25)**		
	**α-diversity**	**β-diversity**		**α-diversity**	**β-diversity**		**α-diversity**	**β-diversity**	
		p-value	R^2 value		p-value	R^2 value		p-value	R^2 value
CD4 (cells/mm^3)	0.427	0.356	0.021	0.446	0.413	0.041	0.550	0.159	0.054
BMI (kg/m^2)	0.416	0.284	0.964	0.462	-	-	0.437	0.991	0.948
Current Tobacco Smoker	0.578	0.495	0.040	0.267	0.444	0.042	0.890	0.674	0.040
Alcohol	0.242	0.434	0.019	0.907	0.985	0.023	0.274	0.245	0.050
INH prophylaxis	0.195	0.115	0.030	0.340	0.103	0.064	0.445	0.897	0.027
Co-trimoxazole prophylaxis	0.665	0.191	0.026	0.193	0.086	0.062	0.946	0.581	0.035
Field site	0.476	**0.001**	0.255	0.201	**0.001**	0.339	0.395	**0.002**	0.284

Supplementary Figure S3: The DMM LaPlace approximation. Three clusters were identified as the best fit using Dirichlet-Multinomial Mixtures (DMMs) in the stool samples collected in the overall cohort.

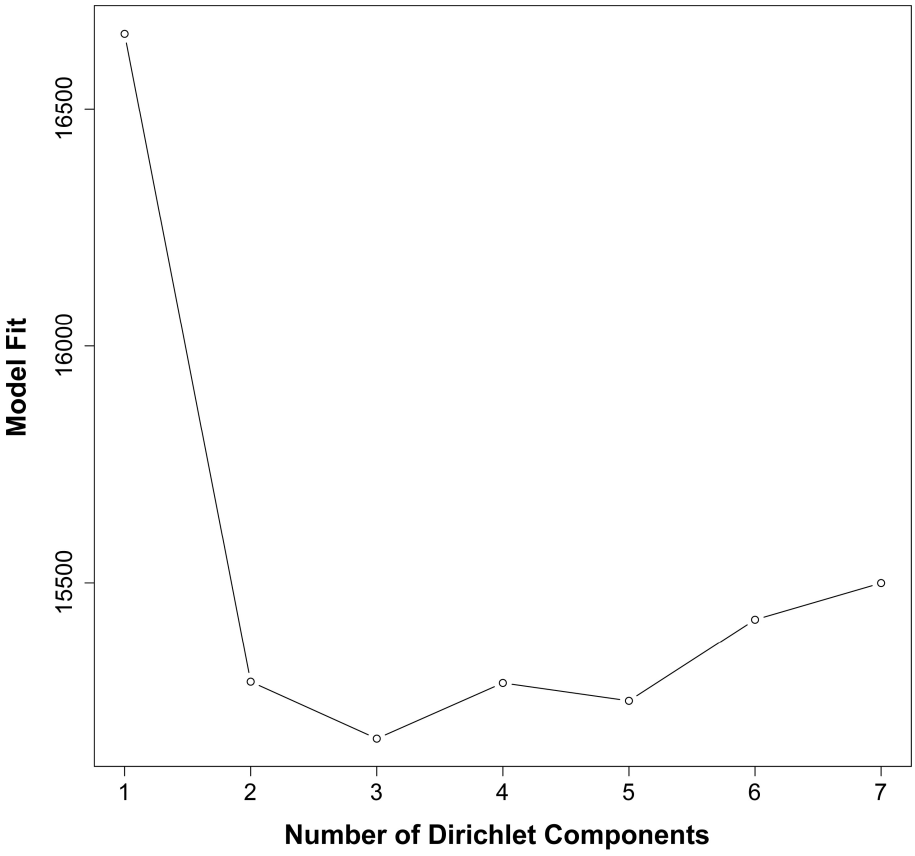

Supplementary Figure S4: Taxonomic differences between the three clusters found in the cohort. (A) Linear discriminant analysis (LDA) effect size (LEfSe) identified significant taxonomic differences in microbiome enrichment based on clusters. (**B**) Relative abundance of each genus.

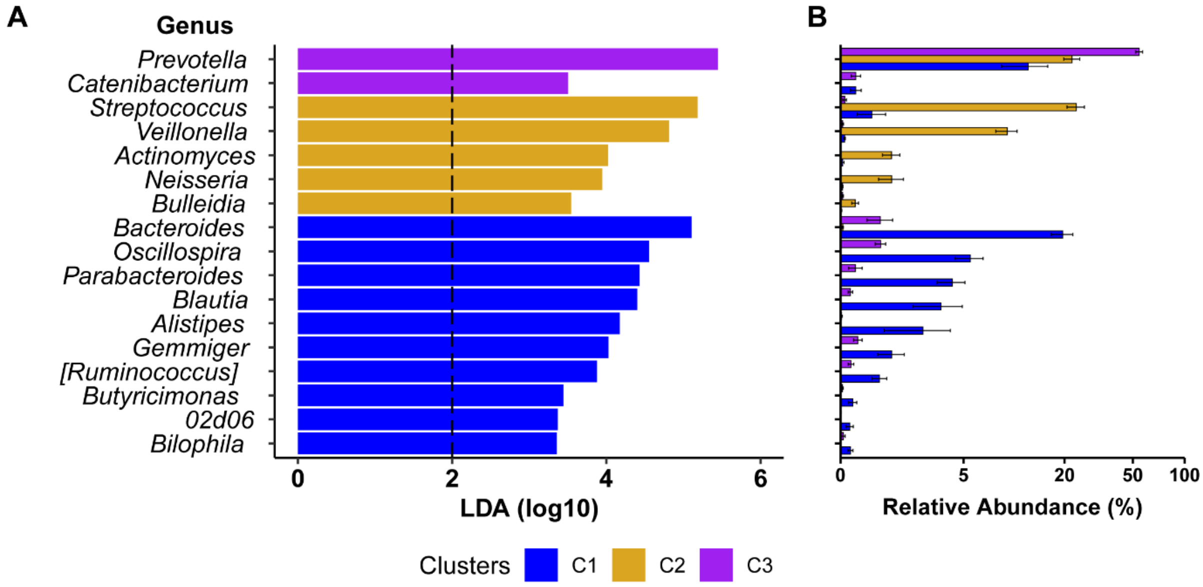

Supplementary Figure S5: No difference between taxa associated with quantitative response to antigen stimulation. (A) Volcano plot depicting differentially abundant taxa based on the median IGRA quantitative response to antigen (median value 0.43). More discriminatory taxa appear closer to the left or right and higher above the threshold (red dotted line, FDR=0.20). (B) Volcano plot depicting differentially abundant taxa based on the median TST response (Median value 25). LTBI: latent TB infection.

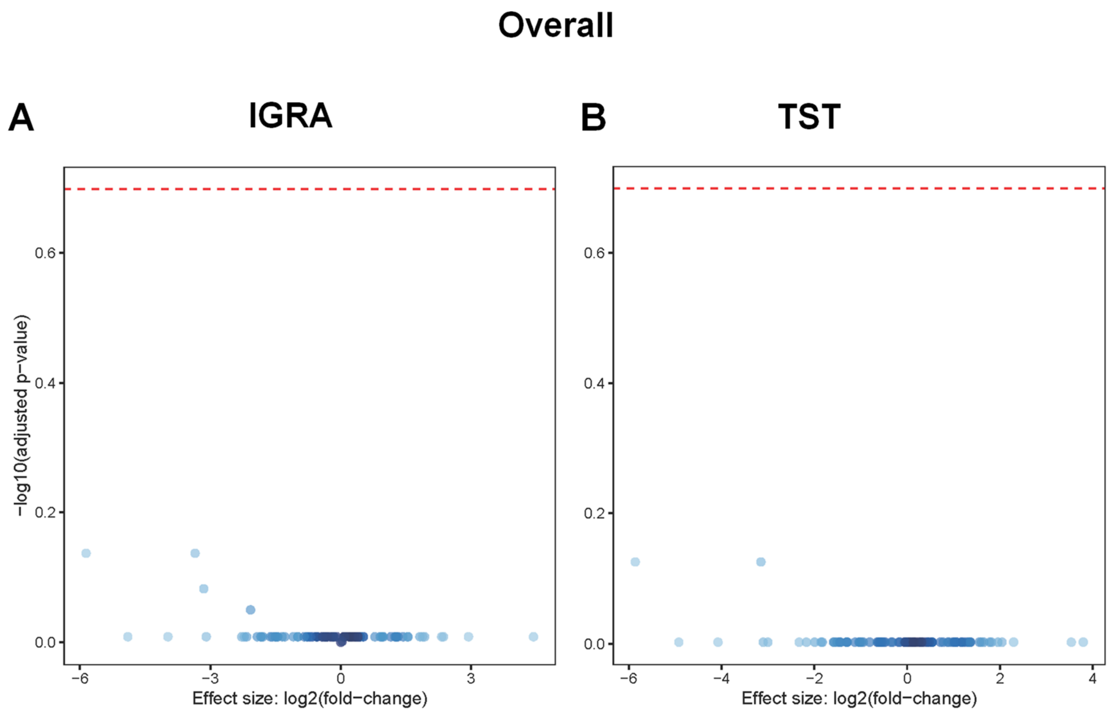

Supplement: Supplementary file 1 [file microorganisms-12-01048-s001.zip › microorganisms-2986537-supplementary.pdf]
